# Poly ε-Caprolactone Nanoparticles for Sustained Intra-Articular Immune Modulation in Adjuvant-Induced Arthritis Rodent Model

**DOI:** 10.3390/pharmaceutics14030519

**Published:** 2022-02-26

**Authors:** Ekta Singh, Riyaz Ali M. Osmani, Rinti Banerjee, Amr Selim Abu Lila, Afrasim Moin, Khaled Almansour, Hany H. Arab, Hadil Faris Alotaibi, El-Sayed Khafagy

**Affiliations:** 1 Department of Biosciences and Bioengineering, Indian Institute of Technology Bombay, Mumbai 400076, India; rinti@iitb.ac.in; 2 Department of Pharmaceutics, JSS College of Pharmacy, JSS Academy of Higher Education and Research, Mysuru 570015, India; riyazosmani@gmail.com; 3 Department of Pharmaceutics and Industrial Pharmacy, Faculty of Pharmacy, Zagazig University, Zagazig 44519, Egypt; a.abulila@uoh.edu.sa; 4 Department of Pharmaceutics, College of Pharmacy, University of Hail, Hail 81442, Saudi Arabia; a.moinuddin@uoh.edu.sa (A.M.); kh.almansour@uoh.edu.sa (K.A.); 5 Department of Pharmacology and Toxicology, College of Pharmacy, Taif University, P.O. Box 11099, Taif 21944, Saudi Arabia; h.arab@tu.edu.sa; 6 Department of Pharmaceutical Sciences, College of Pharmacy, Princess Nourah Bint Abdulrahman University, P.O. Box 84428, Riyadh 11671, Saudi Arabia; Hfalotaibi@pnu.edu.sa; 7 Department of Pharmaceutics, College of Pharmacy, Prince Sattam Bin Abdulaziz University, Al-kharj 11942, Saudi Arabia; e.khafagy@psau.edu.sa; 8 Department of Pharmaceutics and Industrial Pharmacy, Faculty of Pharmacy, Suez Canal University, Ismailia 41552, Egypt

**Keywords:** drug delivery, nanotherapeutics, rheumatoid arthritis, leflunomide, polymeric nanoparticle, poly-ε-caprolactone, adjuvant induced arthritis, intra-articular drug delivery

## Abstract

Rheumatoid arthritis (RA) is a chronic inflammatory autoimmune disorder with synovitis and articular pathology as its primary expositions. Leflunomide (Lfd) is an anti-rheumatic drug that is effective in the treatment of RA, but displays severe side effects upon prolonged systemic administration. Local therapy might represent a promising strategy to treat rheumatoid arthritis without eliciting systemic adverse effects. In this study, leflunomide-loaded poly(ε-caprolactone) nanoparticles (Lfd-NPs) were prepared and assessed as a local drug delivery system capable of alleviating RA-associated inflammation. Lfd-NPs were optimized using the Quality by Design (QbD) approach, applying a 3^2^ full factorial design. In vitro drug release from NPs was examined in simulated synovial fluid. In addition, the in vivo efficacy of Lfd-NPs was evaluated in the Adjuvant Induced Arthritis (AIA) rodent model. Sustained drug release in simulated synovial fluid was observed for up to 168 h. A gradual reduction in paw volume and knee diameter was observed over the course of treatment, indicating the regression of the disease. In addition, significant reductions in serum proinflammatory markers and cytokines, including the C-reactive protein (CRP), rheumatoid factor (RF), TNF-α, IL1-β, and IL-6, were verified upon treatment with Lfd-NPs, suggesting the modulation of immune responses at the pathological site. Most importantly, no remarkable signs of toxicity were observed in Lfd-NP-treated animals. Collectively, intra-articularly administered Lfd-NPs might represent a potential therapeutic alternative to systemically administered drugs for the treatment of rheumatoid arthritis, without eliciting systemic adverse effects.

## 1. Introduction

Rheumatoid arthritis (RA) is an autoimmune disorder that affects the synovial membrane that coats and protects the articular structures of the body [1]. It represents one of the leading causes of chronic morbidity in high–income countries [2]. The prevalence of RA varies from country to country, with an average of 1% in the adult population worldwide [3]. Furthermore, it is more prevalent among women in developed countries, with an approximate male–female incidence ratio of 1:3, indicating higher risk in women than men [4]. The most common symptom in rheumatoid arthritis patients is joint pain, and it is usually associated with physical impairment, reduced mobility, depression, sleep difficulties, and higher healthcare expenses [5,6,7].

Current rheumatoid arthritis treatments, including non-steroidal anti-inflammatory drugs (NSAIDs), corticosteroids (SAIDs), and analgesics, are largely targeted at symptomatic alleviation of joint pain and may only have a relatively little impact on the underlying cascade of joint degeneration [8]. Another important class of therapeutics is disease-modifying antirheumatic drugs (DMARDs), such as methotrexate, leflunomide, sulfasalazine, and hydroxychloroquine [9,10]. They act to alleviate pain and inflammation, minimize or prevent joint deterioration, and preserve joint structure and function via suppressing the body’s overactive immune and/or inflammatory systems [11]. Leflunomide (Lfd), N [(4-trifluoromethyl) phenyl]-5-methylisoxazole-4-carboxamide, is a synthetic isoxazole derivative that possesses anti-inflammatory as well as immunosuppressive properties, and has been licensed as an anti-arthritic drug by The United States Food and Drug Administration (USFDA) [12,13]. It belongs to class II in the Biopharmaceutics Classification System (BCS) [14]. Leflunomide acts by inhibiting the synthesis of DNA, which is crucial for the replication of cells such as those in the immune system. By suppressing the immune system, Lfd can efficiently minimize the inflammation that causes rheumatoid arthritis’s discomfort and swelling [15]. Nevertheless, many reports demonstrated that extensive systemic use of Lfd could trigger severe side effects such as hepatotoxicity, myelosuppression, lung disease, and heart attack [16,17,18,19]. In a case review, 49 cases who received Lfd for a prolonged 7-year period were reported to have hepatic impairment, including 14 fatalities. As a consequence of this study, the FDA had introduced indications of the risk of severe hepatic injury for Lfd [20].

It is worth noting that despite the availability of various drugs for treating RA, systemic administration of such drugs may not produce therapeutic effects in the joint area. This might be attributed to the rapid clearance, the short biological half-lives, and, most importantly, the relatively slow and inadequate transport of drug molecules to joint tissues, which collectively lead to multiple administrations of higher drug doses to achieve the desired therapeutic effect [21]. High and frequent doses of these drugs and their exposure to various organs are reported to be associated with extra-articular side effects and toxicity. Additionally, this makes the treatment expensive [22]. Instead, an increasing interest turns toward the intra-articular drug delivery route, rather than systemic drug administration, for the treatment of degenerative joint disease [23,24,25]. The intra-articular route of administration shows the ability to target drugs to the affected tissues, and thereby could alleviate the side effects associated with systemically administered drugs [26]. Recent studies have demonstrated the positive effect of the intra-articular injection of therapeutic agents such as corticosteroid [27], hyaluronic acid [28], tumor necrosis factor antagonist (infliximab) [29], mesenchymal stem cell (MSC) [30], and platelet-rich plasma (PRP) [31] in reducing cartilage destruction, joint swelling, and inflammation in RA patients. 

Nevertheless, currently available intra-articular drugs often require multiple injections, which impose a large cost burden, reduce patient quality of life, and might increase the risk of complications [32]. Furthermore, a key determinant for the success of intra-articular therapy is the ability to achieve higher intra-articular drug concentrations and prolonged residence times following a local injection to the joint region, as well as lower systemic exposure to the drug. Many parameters, including vehicle composition, particle size, hydrophobicity, and charge, can influence the efficacy of a drug delivery vehicle in promoting a sustained intra-articular residence time.

The development of nanoparticle-based drug formulations has opened up new avenues for addressing and treating challenging diseases. The entrapment of active pharmaceutical ingredients within nanoparticulate systems, such as nanoparticles and liposomes, could improve the pharmacokinetics and biodistribution of the drug molecules, and could thereby enhance the therapeutic efficacy of the entrapped drug while minimizing drug-related side effects [33]. In addition, by virtue of the enhanced permeation and retention (EPR) effect, nanoparticulate systems can exploit the leaky nature of blood vessels in inflamed areas for achieving a more site-specific delivery of therapeutic agents [34]. Furthermore, decorating the surface of nanocarriers with ligands against certain cellular receptors being over-expressed in RA might offer the chance for achieving active drug targeting [35]. Biodegradable polymeric drug delivery devices comprised of poly(amides), poly(anhydrides), polyesters, poly(ortho esters), poly(alkyl cyanoacrylates), poly(ester amides), and numerous other polysaccharides have been approved long ago by the US FDA for clinical applications [36]. Poly(ε-caprolactone) (PCL) is one such FDA-approved biodegradable polymer; it has been extensively investigated and applied in developing new-fangled drug delivery systems as an implantable biomaterial, and is the preferred polymer in designing injectable implant-based controlled-release drug delivery systems [37,38,39,40]. Nonetheless, owing to its slow eroding and hydrophobic nature, PCL has been widely explored in designing and developing nanocarrier-based intra-articular delivery systems, as well as drug-eluting long-acting implants [38,39,40,41]. In this context, local intra-articular drug delivery can be adequately used to target inflamed sites, specifically for direct immune suppression and the reduction of systemic biodistribution and extra-articular toxicity.

The aim of the current study was, therefore, to formulate and optimize poly-ε-caprolactone nanoparticles loaded with the anti-rheumatic drug leflunomide for local intra-articular administration, using a two-factor, three-level full factorial design. The optimized formula was evaluated for its physicochemical characteristics, in vitro release and in vitro cytotoxicity levels, and biocompatibility. In addition, the in vivo efficacy of the formulated nanoparticles in modulating the inflammation was investigated in an adjuvant-induced arthritis (AIA) rat model. 

## 2. Materials and Methods

### 2.1. Materials

Leflunomide (Lfd) was a gift sample from Cipla Ltd. (Mumbai, India). Poly ε-caprolactone (average Mw ~14,000, average Mn ~10,000 by GPC), polyvinyl alcohol (average Mw ~130,000 g/mol, 99+% hydrolyzed, HLB value ~18), hyaluronic acid, 3-(4,5-dimethylthiazol-2-yl)-2,5-diphenyl tetrazolium bromide (MTT), phorbol 12 myristate 13-acetate (PMA), lipopolysaccharides (LPS), bovine Serum Albumin (BSA), Bradford reagent, Freund’s Complete Adjuvant (FCA) were purchased from Sigma Aldrich-Merck Ltd. (Mumbai, India). Dulbecco’s Modified Eagle’s Medium (DMEM), Roswell Park Memorial Institute medium (RPMI 1640), fetal bovine serum (FBS), trypsin EDTA solution, and Hanks’ Balanced Salt Solution was procured from HiMedia (Mumbai, India). All other reagents and solvents procured were of analytical grade and used as per manufacturer’s instructions. 

### 2.2. Preparation of Leflunomide-Loaded Nanoparticles (Lfd-NPs)

Leflunomide-loaded nanoparticles (Lfd-NPs) were prepared by a bottom-up approach, commonly known as the nano-precipitation technique [42]. Briefly, the aqueous phase was prepared by dissolving an adequate quantity of polyvinyl alcohol (PVA), which acts as a surfactant [43,44], in 25 mL distilled water. The organic phase was prepared by dissolving poly ε-caprolactone in 12.5 mL acetone using bath sonication until a clear solution was visible. Lfd (in a drug–polymer weight ratio of 1:2) was added to the organic phase. The organic solution of the polymer containing Lfd was then added drop-wise via a needled syringe and left overnight for stirring. The formed NPs were stored at an ambient temperature for further studies.

### 2.3. Experimental Design

Experimental design, a systematic as well as scientific approach, was used to explore the relationship and interaction among the independent and dependent variables. A two-factor, three-level (3^2^) full factorial design, using Design Expert^®^ (version 12.0.3.0, Stat-Ease Inc., Minneapolis, MN, USA), was adopted for optimizing leflunomide-loaded nanoparticles (Lfd-NPs), and to explore the effect of different formulation variables, namely poly ε-caprolactone (PCL) concentration (A) and polyvinyl alcohol (PVA) concentration (B), on product characteristics, namely particle size (R1), entrapment efficiency (R2), and in vitro drug release (R3). The optimization of the PCL (A) and PVA (B) concentrations for the preparation of Lfd-NPs was obtained by incorporating a Design of Experiment (DoE) at three levels: high (+1), medium (0), and low (−1) (Table 1). A total of nine runs were prepared (Table 2). In order to assess the formulation responses, a statistical model introducing interactive and polynomial terms was employed, given by the equation below:

R = b_0_ + b_1_A + b_2_B + b_3_AB + b_4_A^2^ + b_5_B^2^

In the above equation, R represents the response, and b_0_ is the arithmetic mean response of the nine runs. The responses in the above equation comprise the quantitative influence of the independent variables A and B; b_1_, b_2_, b_3_, b_4_, and b_5_ are the estimated coefficients for the factors A and B. Table 1 illustrates the details of the factorial design applied for the optimization.

### 2.4. Structural Characterization of Lfd-NPs

#### 2.4.1. Fourier Transform Infrared Analysis 

Fourier transform infrared (FTIR) spectroscopy (4700, Jasco, Tokyo, Japan) was used to detect any interaction between Lfd and nanoparticle excipients. The spectra were recorded within the wave number range 4000 cm^−1^ to 400 cm^−1^.

#### 2.4.2. Drug Crystallinity Study

X-ray powder diffractograms of the pure drug and excipients in their pure form, as well as physical mixtures of various combinations of drug and excipients and drug-loaded nanoparticles, were verified using an X-ray diffractometer (Smartlab, Rigaku, Tokyo, Japan). Diffraction patterns were obtained in the interval 2θ = 0–90° using Ni-filtered Cu K(α) radiation (~1.54 A), 45 kV° voltage, 40 mA current with a scan speed 0.01 s^−1^.

#### 2.4.3. Differential Scanning Calorimetry (DSC) Analysis

Differential scanning calorimetry (DSC 60 Shimadzu, Tokyo, Japan) was conducted for Lfd, the physical mixture, blank (empty) nanoparticles, and optimized Lfd-NPs. A high-purity alumina disc (empty cell) was used as the reference. For instrument calibration, high-purity indium metal was used as a standard. Dynamic scans were performed under a nitrogen atmosphere at temperatures ranging from 10–300 °C and a heating rate of 10 °C per minute. 

### 2.5. Evaluation of Prepared Nanoparticles

#### 2.5.1. Particle Size and Zeta Potential Analysis

The hydrodynamic particle size of formulated nanoparticles was analyzed by dynamic light scattering (DLS) technique. Malvern Instrument (Zetasizer Ver. 7.11, Malvern, UK) was adopted to estimate the size, surface charge, and particle size distribution of the prepared nanoparticles. The experiments were performed in triplicates using a clear disposable zeta cell, and using water as a dispersant at 25 °C.

#### 2.5.2. Microscopic Imaging

The morphology and surface topography of the prepared leflunomide-loaded nanoparticles (Lfd-NPs) were imaged by field emission gun-based scanning electron microscopy (FEG-SEM) in Cryo mode (JSM 7600F, JEOL Ltd., Tokyo, Japan), operating at an acceleration voltage range of 0–25 kV and suitable magnification. Briefly, samples were loaded onto metal stubs and frozen with slush nitrogen. Sputter coating was performed with platinum under an argon atmosphere. 

#### 2.5.3. Production Yield

Lfd-NP suspensions were subjected to 30 min centrifugation at 20,000 rpm, the obtained pellet was re-dispersed in cryoprotectant solution (mannitol 5% *w*/*v*) and freeze dried for 48 h at −55 °C and vacuum < 50 mTorr, using a ScanVac CoolSafe freeze dryer (LaboGene ApS, Lynge, Denmark), to obtain dried free-flowing NP mass. These freeze-dried NPs were precisely weighed and the production yield was calculated using the following formula [45]:

$$\mathrm{Production}\;\mathrm{yield}\;(\%)=\frac{Practical\;mass\;of\;nanoparticles}{Theoretical\;mass\;of\;nanoparticles\;\left(polymer+drug\right)}\times 100$$

#### 2.5.4. Drug Entrapment Efficiency 

The drug entrapment efficiency was quantified by reverse phase high performance liquid chromatography with a photodiode array (PDA) detector (RP-HPLC, MD 4015, Jasco, Tokyo, Japan). The chromatographic separation was conducted using an isocratic elution. A mixture of acetonitrile and water (40:60) was used as mobile phase. The separation was carried out using a Li Chrosphere C18 column (Agilent Technologies, Santa Clara, CA, USA). An injection volume of 20 μL was used with a flow rate of 1 mL/min. Detection of standard peak was carried out at 262 nm for Lfd at a retention time of 7.3 min. Drug dilutions were prepared in methanol and a standard curve was plotted by calculating the peak area for each concentration. For the determination of drug entrapment efficiency, Lfd-NPs were dissolved in methanol, diluted suitably with methanol, and then injected in HPLC for quantification of the entrapped drug. The following equation was used to calculate encapsulation efficiency [46].

$$\mathrm{Entrapment}\;\mathrm{efficiency}\;(\%)=\frac{Entrapped\;drug\;amount}{Total\;drug\;amount}\times 100$$

### 2.6. In Vitro Drug Release

In vitro release of leflunomide from developed Lfd-NPs was monitored in simulated synovial fluid (SSF, pH 7.4; Table S1) using classic dialysis method [47]. A dialysis membrane with size exclusion limit of 50 KDa (HIMEDIA^®^ LA 387 Dialysis Membrane-50, HIMEDIA, Mumbai, India) was used. Briefly, a definite weight of Lfd-NPs was sealed in dialysis tubing and was suspended in 50 mL simulated synovial fluid (SSF, pH 7.4; Table S1) at 37 °C under slow, constant stirring at 20 rpm. At different time intervals up to 168 h, 1 mL aliquots of the SSF were withdrawn, with simultaneous replenishment with 1 mL of fresh SSF to maintain the sink condition. The amount of Lfd released was quantified using RP-HPLC as aforementioned.

### 2.7. In Vitro Cellular Studies

#### 2.7.1. Cell Lines 

L929 murine fibroblast (ATCC^®^ CCL-1^™^), THP1 human monocyte (ATCC^®^ TIB-202^™^), Jurkat T (ATCC^®^ TIB-152^™^), and RAW 264.7 murine macrophage (ATCC^®^ TIB-71^™^) cell lines were purchased from National Centre for Cell Science (NCCS, Pune, India). L929 and RAW 264.7 cells were maintained in an adherent culture flask in complete Dulbecco’s Modified Eagle’s Medium (DMEM). THP1, and Jurkat T cells were maintained as a suspension culture in Roswell Park Memorial Institute medium (RPMI 1640). DMEM and RPMI 1640 medium were supplemented with 10% fetal bovine serum (FBS) and 1% antibiotic-antimycotic solution. Both the cultures were incubated at 37 °C in 5% CO_2_ and 70% relative humidity in an incubator.

#### 2.7.2. In Vitro Biocompatibility

Serial dilutions of leflunomide-loaded nanoparticles (Lfd-NPs) were prepared in DMEM with concentrations ranging from 5 to 1000 ng/mL. L929 cells were seeded into a 96-well plate with a cell density of 1 × 10^4^ cells/well, and were incubated for 24 h under standardized conditions. After the specified incubation time, spent media were removed and cells were treated with different concentrations of Lfd-NPs, followed by 48 h incubation. After incubation, spent media were discarded and cells were treated with 100 μL MTT (1 mg/mL in Hanks balanced salt solution) and further incubated for 3 h. At 3 h post-incubation, MTT solution was removed from cells and 200 μL of DMSO was added to dissolve the formazan crystals formed by MTT. Cell viability was then calculated by measuring absorbance at 560 nm using ERBA Lisa Scan EM Plate reader (Erba Mannheim, Mumbai, India).

#### 2.7.3. In Vitro Cellular Uptake

THP 1 cells (1 × 10^7^ cells/well) were seeded in the presence of 0.1% phorbol 12-myristate 13-acetate (PMA) on a cover-slip in a 24 well plate and incubated at 37 °C for 72 h. PMA was added to stimulate the cells’ differentiation into macrophage cells. At 72 h post-incubation, spent media were discarded and replaced with 1 mL fresh DMEM containing 100 μg/mL nanoparticles loaded with rhodamine-6-G (R6G). The cells were further incubated for 3 h. At 3 h post-incubation, the spent media was discarded and the cells were rinsed trice with cold PBS (pH 7.4), fixed with 10% formaldehyde, and then rinsed again trice with cold PBS (pH 7.4). The cover-slips were glycerol mounted on glass slides and visualized under 63X objective in confocal laser scanning microscope using excitation-emission wavelengths of 524/547 nm. 

#### 2.7.4. Evaluation of In Vitro Anti-Inflammatory and Immunosuppressive Effects of Lfd-NPs 

To study the anti-inflammatory and immunosuppressive effects of Lfd-NPs, THP1 human monocytes, RAW 264.7 murine macrophages, and Jurkat T cell lines were used. THP1 and RAW 264.7 cells were seeded in 96-well plates at a cell density of 1 × 10^5^ cells/well. THP1 cells were exposed to PMA at a concentration of 10 ng/mL for 48 h to stimulate their differentiation into macrophage cells. Non-adherent macrophage cells were washed off using PBS. Injury to the cells was caused by lipopolysaccharides (LPS, 2 μg/mL) for 2 h, then the cells were immediately treated with either free Lfd or Lfd-NPs. Injury-induced, untreated cells were taken as positive control. Supernatant was collected after 24 h interval and tested using DuoSet^®^ ELISA kit (R&D Systems, Wiesbaden, Germany) for IL-6 and TNF-alpha as per manufacturer’s protocol. Absorbance was taken at 450 nm using the ERBA Lisa Scan EM Plate reader. Reference curve was obtained by plotting concentration of standard protein (BSA). Jurkat cells were seeded at a density of 0.5 × 10^5^ cells/well in triplicates. The cells were treated with either free Lfd or Lfd-NPs at a concentration of 10 ng/mL. Untreated and DMSO-treated cells were taken as controls. Anti-proliferation activity and cell viability was assayed by Trypan blue dye exclusion assay at different time points (0, 24, 48 and 72 h).

### 2.8. In Vivo Studies

#### 2.8.1. Animals

Male Wistar albino rats (6–5 weeks old, 200–300 g) were supplied by Biogen Laboratory Animal Facility (Bengaluru, India). The animals were maintained in a temperature- and humidity-controlled environment, and were fed standard laboratory chow and water ad libitum. All animal experiments were approved by the Institutional Animal Ethical Committee (IAEC) for APT Testing and Research Pvt. Ltd., Pune, India, following the CPCSEA Guidelines (approval No. RP20/1920). 

#### 2.8.2. Adjuvant-Induced Arthritis Rodent Model 

The adjuvant-induced arthritis (AIA) model is one of the most commonly used standard arthritis models [48]. In this study, Wistar albino rats were divided into 4 groups (n = 6 each), namely: normal control (NC), diseased control (DC), treated with oral Lfd (LO), and treated with intra-articular Lfd-NPs (Lfd-NP). To induce arthritis, rats were intra-articularly injected with 0.1 mL of Complete Freund’s Adjuvant (CFA) in the right knee. A booster dose of CFA was given similarly on the 7th day of initial induction. From the 14th day after the initial induction, LO group was treated daily with an oral suspension of commercial leflunomide (10 mg/kg). Daily oral administration of Lfd was adopted to mimic the administration frequency in clinical settings, while, Lfd-NP group was injected with Lfd-NPs intra-articularly (10 mg/kg) once/week. To evaluate treatment efficacy, body weight was measured weekly, whereas paw volume and knee diameter were measured at regular short intervals. Furthermore, the animals were euthanized at the end of the treatment schedule and serum samples were analyzed for C-reactive protein (CRP), Rheumatoid factor (RF), Tumor necrosis factor alpha (TNF-α), interleukin 1β (IL-1β), and interleukin 6 (IL-6) using RayBio^®^ ELISA kit (Raybiotech, Norcross, GA, USA). Joint tissues were also isolated from animals and then stored in 10% formalin solution for fixation. The samples were processed to make a paraffin block, which was then sectioned (5 μm), and were subjected to haematoxylin and eosin (H&E) staining. 

#### 2.8.3. Toxicological Studies of Lfd-NPs

In order to verify the safety of different treatments, the animals were euthanized at the end of study; vital organs (liver, kidney, lung, heart) were dissected and duly weighed. Histopathological analysis of paraffin embedded tissue blocks and biochemical analysis of blood were performed to check for any abnormality or toxicity. 

### 2.9. Statistical Methods

All the experiments were carried out in triplicates and the results obtained are represented as mean ± standard deviation. Statistical significance was determined using Graph pad prism 7 version by students t–test and one way and two-way analysis of variance (ANOVA), where *p* < 0.05 was considered to be statistically significant. 

## 3. Results

### 3.1. Formulation of Leflunomide-Loaded Nanoparticles (Lfd-NPs)

#### 3.1.1. Full Factorial Design Experiment and Response Surface Analysis

Factorial design is a research approach that allows for the analysis of the main and interaction effects of two or more independent factors on one or more outcome variables. In this study, leflunomide-loaded nanoparticles (Lfd-NPs) were formulated and optimized by a two-factor, three-level (3^2^) full factorial design. A total of nine batches of formulations, namely F-1 to F-9, were prepared by modifying two independent variables, A (PCL concentration) and B (PVA concentration), and their impact on three formulation variables, namely, particle size (R1), entrapment efficiency (R2), and cumulative drug release (R3), were explored. The design matrix of the variables and responses is summarized in Table 2. Multiple regression analyses were performed on the responses selected for optimization in order to develop polynomial equations whose coefficient values reflected the influence of individual variables, and contour as well as 3D response surface plots were created.

##### Effect of Formulation Variables on Particle Size of Lfd-NPs

The efficiency of a nanocarrier is critically affected by their particle size. There is a significant relation between particle size and the rate and extent of drug release, as well as drug absorption. The prepared NPs were noted to exhibit a uniformity in size devoid of any polydispersion. The particle sizes were noted as being in the range of 169 ± 19.5 to 754 ± 11.9 nm. The quantitative effects of independent variables (A and B) on particle size (R1) were fitted into a regression analysis, and a second-order polynomial equation was derived to describe the mathematical relationships between the dependent and independent variables:

R1 = +260.44 + 245A + 8.33B + 8.5AB + 131.33A^2^ + 95.33B^2^

**Figure 1:**
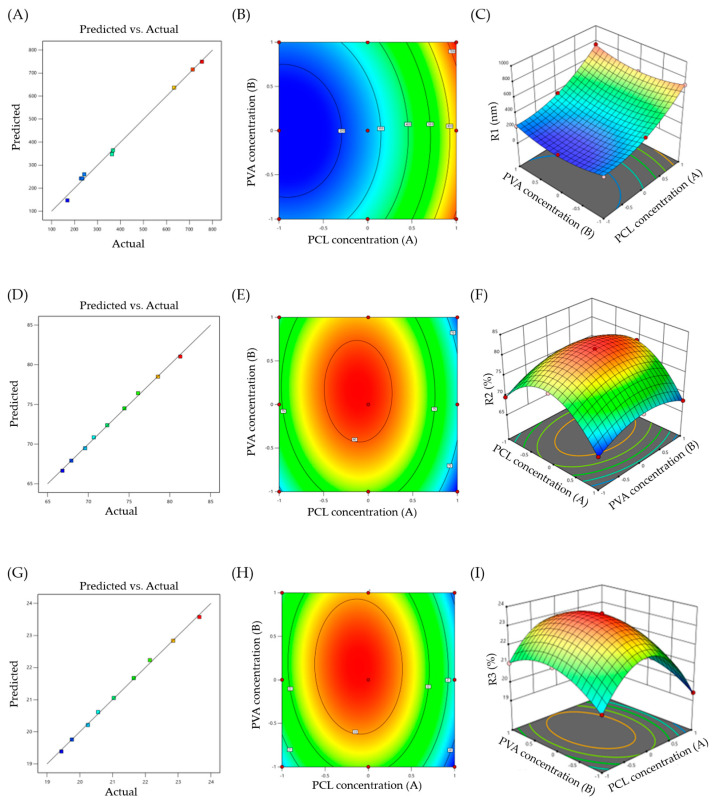
(**A**) Response surface plots of particle size (R1); (**B**) Contour plot of R1; (**C**) 3D surface plot for R1; (**D**) Response surface plots of entrapment efficiency (R2); (**E**) Contour plot of R2; (**F**) 3D surface plot for R2; (**G**) Response surface plots of cumulative drug release (R3); (**H**) Contour plot of R3; (**I**) 3D surface plot for R3.

Analysis of variance (ANOVA) was adopted to justify the adequacy and significance of the model. ANOVA results revealed that the model was significant, with an F value of 178.15 and *p* value < 0.0001. The positive or negative sign in the polynomial terms indicated the effect of the different levels of combinations of the independent variables on the responses. Figure 1A supports the linearity of the data, where the adjusted R^2^ value of 0.991 was in correlation with the predicted one (0.9678). Figure 1B,C shows 2D contour and 3D response surface plots that depict the impact of the independent variables (A and B) on the R1 response, respectively. It was obvious that the particle size of the prepared nanoparticles is directly affected by changes in polymer concentration. At a fixed PVA concentration, an increasing polymer concentration significantly increased the particle size from 169 ± 19.5 to 633 ± 22.1 for F-3 and F-9. Similar results were reported by Dandagi et al., who demonstrated that an increasing polymer concentration resulted in a significant increase in the particle size of mebeverine-loaded microspheres [49]. 

##### Effect of Formulation Variables on Entrapment Efficiency of Lfd-NPs

In addition to the particle size, optimum entrapment efficiency is another prerequisite for developing an effective intra-articular drug delivery system. A high entrapment efficiency is required to maintain drug concentration, which is important for promoting prolonged localized drug release in the synovial cavity. The results of the entrapment efficiency analysis indicated efficient drug loading within NPs. The entrapment efficiency of all the formulations lied in the range of 66.83 ± 3.2% to 81.29 ± 2.1% (Table 2). By applying a 3^2^ full factorial design, different responses for entrapment efficiency (R2) were observed at different combinations of the factors A and B. The following polynomial equation was obtained from the best fit of the response R2 to the independent variables:

R2 = + 81.03 − 1.83A + 1.04B − 0.4075AB − 8.35A^2^ − 3.57B^2^

ANOVA of the equation gave a model F value of 451.66, and a *p* value < 0.05, suggesting that the model was significant. In addition, the predicted R2 of 0.9876 was in reasonable agreement with the adjusted R^2^ of 0.9965 (Figure 1D). Figure 1E,F depict 2D contour and 3D response surface plots that imply the effect of the independent variables (A and B) on the R2 response, respectively. It was evident that increasing the concentrations of the two independent variables (A and B) led to an increase in the entrapment efficiency, presumably due to the mutual increase in particle size with an increasing polymer concentration. 

##### Effect of Formulation Variables on In Vitro Drug Release from Lfd-NPs

In vitro drug release studies provide essential information for predicting the in vivo performance of the formulation. A prolonged drug release from NPs is a key determinant of the efficacy of Lfd following intra-articular administration. In this study, a prolonged drug release for up to seven days was observed in the in vitro release profile. The following equation was obtained to elucidate the correlation between R3 and the two independent variables by the best-fit mathematical model:

R3 = + 23.57 − 0.53A + 0.3033B − 0.1175AB − 2.43A^2^ − 1.04B^2^

The model shows an F value of 445.47, with a *p* value < 0.05, suggesting that the model was significant. The linearity of the data was verified by the linear correlation plot (Figure 1G), where the observed adjusted R^2^ value was 0.9964 and the predicted R^2^ was 0.9875. Figure 1H,I represent the 2D contour and 3D response surface plot for the effect of two independent variables on in vitro drug release, respectively. It was obvious that factor A affected the release response significantly more than factor B. Furthermore, there was an increase followed by a decrease in drug release, with an increase in A and B concentrations. 

#### 3.1.2. Selection of Optimized Formula

A multi-criteria decision approach was deployed for optimizing all the responses with different targets, applying the desirability function for numerical optimization. The desirable responses were set to fulfil the following criteria: particle size in the range of 190–290 nm, maximum drug entrapment efficiency, and maximum drug release. At a desirability value of 0.995, the optimized formula for Lfd-NPs was obtained at a PCL concentration of 99.8 mg and a PVA concentration of 2.2% *w*/*v*. The optimized formula was prepared for checkpoint analysis and evaluated for particle size (nm), entrapment efficiency (%), and cumulative in vitro drug release (%). The observed particle size, entrapment efficiency and cumulative drug release were 240 ± 14.6 nm, 79.8 ± 4.4%, and 23.8 ± 4.3%, respectively, which were close to the predicted values (237.7 nm, 81.213%, and 23.62%) for the optimized formula. These results represented the reliability of the optimization protocol for the preparation of the formulations according to the 3^2^ full factorial designs. 

### 3.2. Characterization of Optimized Lfd-NPs

#### 3.2.1. Particle Size, Zeta Potential, and Poly Dispersity Index

**Figure 2:**
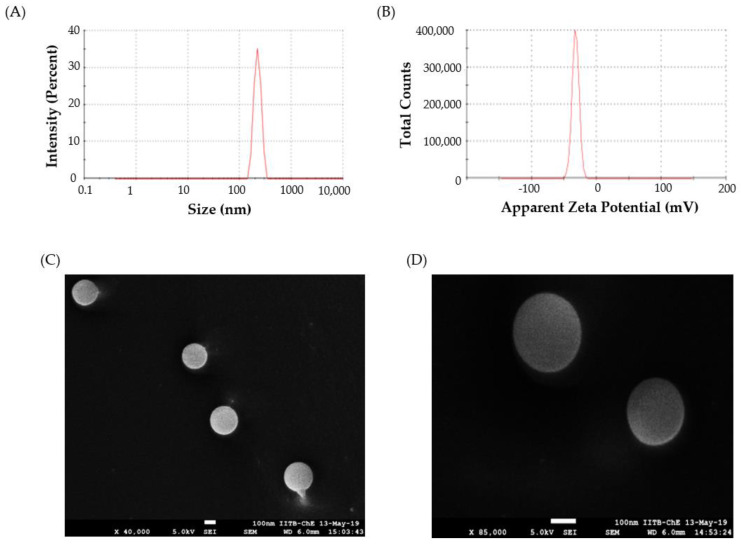
(**A**) DLS size distribution plot; (**B**) Zeta potential for Lfd-NPs; (**C**,**D**) Cryo FEG—SEM images at low (×40,000) and high (×85,000) magnification of Lfd-NPs.

Particle size plays a critical role in determining the rate and extent of drug release, as well as the in vivo fate of a nanoparticle [50]. In this study, the nano-sized polymeric particles were fabricated in order to achieve a prolonged and sustained release of Lfd; however, there is still a chance of a leakage of these particles from the pores in the synovial membrane into the bloodstream [51]. By optimizing the size and properties of the Lfd-NPs, the possibility of a loss of these particles into the bloodstream could be minimized. The mean particle size of the optimized Lfd-NPs was 240 ± 14.6 nm, as assessed by DLS (Figure 2A), which might grant the retention of NPs within the synovial cavity. The other parameter measured using the DLS was the polydispersity index. The polydispersity index is defined as the ratio of the particle’s size deviation to its average diameter, and a higher value indicates large variations in particle size [52]. The PDI of optimized Lfd-NPs was found to be 0.17, indicating a uniform size distribution and monodispersity. 

The zeta potential, a representative of the charges borne by the nanoparticle, is another important parameter that significantly affects nanoparticle stability. The zeta potential reflects the surface charge of the particles, which is influenced by changes in the interface with the dispersing medium, due to the dissociation of functional groups on the particle’s surface or to the adsorption of ionic species present in the aqueous dispersion medium, as well as the solvation effect [53]. Generally, zeta values of ± 30 mV suggest that the the colloidal suspension has good physicochemical stability, as large repulsive forces tend to prevent aggregation due to occasional collisions with adjacent nanoparticles [54]. The zeta potential of optimized Lfd-NPs was −32.5 ± 5.3 mV (Figure 2B), suggesting that the optimized NPs have good stability. 

#### 3.2.2. Morphological Studies

FEG-SEM imaging of the optimized Lfd-NPs in cryo mode revealed that the optimized NP formula has a discrete spherical shape with a nearly smooth surface (Figure 2C,D). It is worth noting that local heating from the SEM beam can alter the morphology of the particle and damage it; however, no such deformation was observed in the morphology of Lfd-NPs.

#### 3.2.3. Production Yield

The production yield of the optimized Lfd-NPs formula was found to be 94.71 ± 2.9%. This value was within the acceptable range, indicating that there was minimum loss of the drug during the synthesis and processing of Lfd-NPs.

#### 3.2.4. Entrapment Efficiency 

The quantitative estimation of the drug encapsulated within the polymeric nanoparticles is a crucial factor, influencing release characteristics as well as therapeutic efficacy. The drug entrapment efficiency of optimized Lfd-NPs was found to be 79.83 ± 4.1, and it was well-correlated with the predicted entrapment of the optimized batch. A high entrapment efficiency is indispensable to maintain adequate drug concentrations at the target site, along with ensuring the prolonged intra-articular release of the drug. 

### 3.3. Drug-Excipients Interaction Studies

#### 3.3.1. X-ray Powder Diffraction (XRPD) 

**Figure 3:**
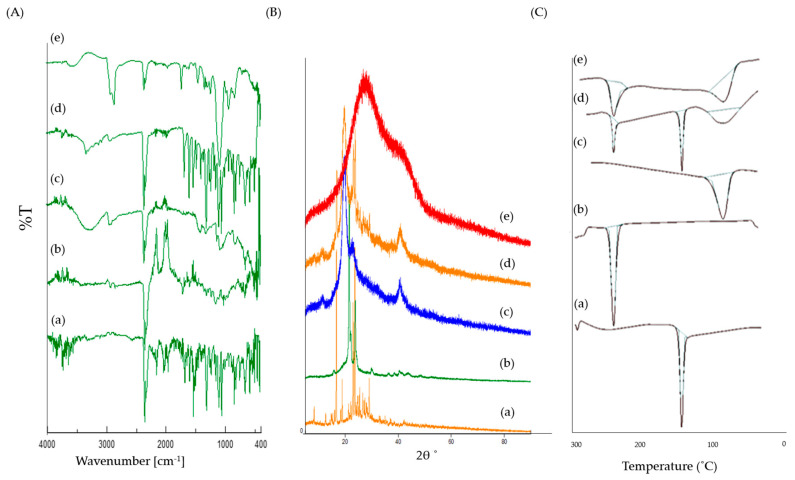
(**A**) FTIR spectra; (**B**) X-ray Diffractograms; (**C**) DSC thermograms for (a) Pure Lfd, (b) Pure PCL, (c) Pure PVA, (d) Physical mixture, (e) Lfd-NPs.

The physical characteristics and purity of the Lfd sample, and its interaction with excipients, were analyzed using the XRPD technique. X-ray diffractograms were obtained for the pure drug as well as Lfd-NPs, as shown in Figure 3A. The XRPD pattern of pure Lfd revealed intense and sharp diffraction peaks at the 2θ values of 16.69°, 18.9°, 22.99°, 23.61°, 29.05°, 29.83°, and 32.95°, suggesting its crystalline nature. On the other hand, the. distinct diffraction peaks of Lfd disappeared in Lfd-loaded nanoparticles. These results suggest the decreased crystallinity in the formulations and/or the drug’s transformation into a non-crystalline state when entrapped within polymeric NPs. 

#### 3.3.2. Fourier Transform Infrared Spectroscopy

The FTIR spectra of pure Lfd, PCL, PVA, and Lfd-NPs are shown in Figure 3B. The FTIR spectrum of the pure drug showed characteristic peaks at 3326.61 cm^−1^ (NH stretch of amide), 2939.95 cm^−1^ (aromatic C−H stretch), 2857.99 cm^−1^ (CH stretching vibration), 1684.52 cm^−1^ (HC = N−O of isoxazole ring), 1604.48 cm^−1^ (C = O of amide), 1538.92 cm^−1^ (C = N stretch of isoxazole ring), 1519.63 cm^−1^ (aromatic C = C stretch), 1485.88 cm^−1^ (C = C stretch of isoxazole ring), 1318.11 cm^−1^ and 1238.08 cm^−1^ (C−H bend in plane), and 939.16 cm^−1^, 822.49 cm^−1^, and 759.81 cm^−1^ (C−H bend out of plane). Of interest, remarkable changes were observed in the FTIR spectrum upon encapsulating Lfd within the nanoparticles. The shifting of some peaks to a higher or a lower wave number, including peaks at 3302.5 cm^−1^, 2922.59 cm^−1^, 2849.31 cm^−1^, 1694.16 cm^−1^, 1631.48 cm^−1^, 1367.28 cm^−1^, 1236.15 cm^−1^, 1018.23 cm^−1^, and 669.178 cm^−1^, was observed. In addition, the disappearance of some peaks in the region of 1538-1485 cm^−1^ was detected. Such alterations suggest the presence of interactions between Lfd and the different constituents used for the formulation of Lfd-NPs.

#### 3.3.3. Differential Scanning Calorimetry

The pure Lfd, PCL, PVA, and Lfd-NPs samples were subjected to thermal analysis, applying the DSC technique. The obtained DSC thermograms are presented in Figure 3C. DSC analysis of pure Lfd depicted a gradual enthalpy change and reproduced a sharp endothermic peak at 165.6 °C, corresponding to its melting point [55]. On the other hand, consistent with the XRPD results, the thermogram of the Lfd-loaded nanoparticles did not show such a characteristic peak, presumably due to the decreased crystallinity in the formulations and/or the drug’s transformation into a non-crystalline state when entrapped within polymeric nanoparticles. These results suggest that the drug was uniformly dispersed at the molecular level in the nanoparticles.

### 3.4. In Vitro Drug Release

**Figure 4:**
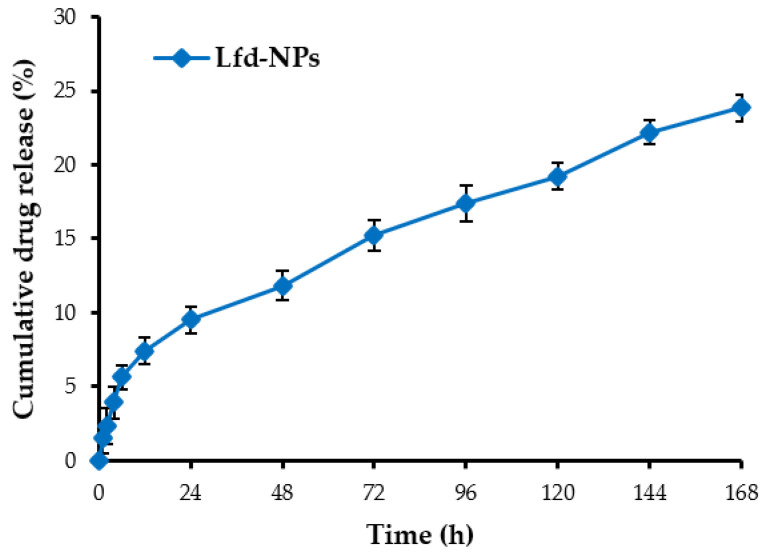
In vitro drug release of leflunomide from Lfd-NPs in simulated synovial fluid at 37 °C.

The in vitro release study of leflunomide (Lfd) from Lfd-NPs was conducted in simulated synovial fluid (SSF, pH 7.4) to demonstrate sustained drug release from the nanoparticles. Figure 4 shows the in vitro release profile of Lfd from optimized Lfd-NPs at different time intervals. It was obvious that Lfd-NPs exhibited a controlled and sustained release of Lfd for seven consecutive days, with only 23.8 ± 4.3% of the drug released from the nanoparticles over the course of 168 h. No burst release of the drug was observed in the release pattern, which confirmed that Lfd was not adsorbed on the NP surface, but was instead well entrapped within the NP. The controlled and sustained release of Lfd from NPs might be attributed to the hydrophobic nature of the drug and polymer. The release data was fitted into various kinetic models. The Higuchi model showed the highest value for r^2^ (r^2^ = 0.92), which indicated that drug release from NPs followed polymer-diffusion-based release kinetics. Poly ε-caprolactone is known to exhibit an extremely slow biodegradation rate, which can be extended up to long durations [56]. This suggests that the mechanism for drug release from polymeric NPs is mainly mediated via drug diffusion, rather than polymeric erosion. 

### 3.5. In Vitro Cellular Studies

#### 3.5.1. In Vitro Cellular Uptake

**Figure 5:**
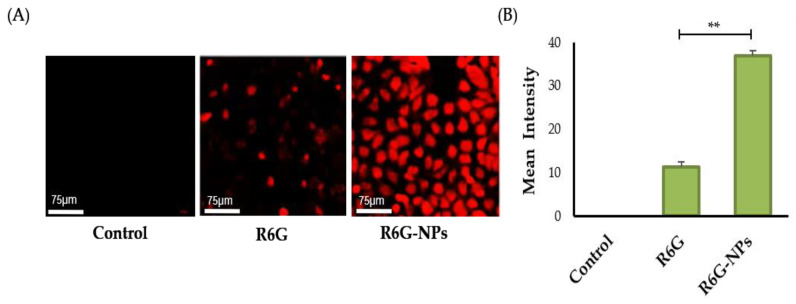
(**A**) Cellular uptake of dye-loaded NPs in THP-1 cell lines using Rhodamine-6-G. Unstained cells and cells treated only with free dye were taken as controls. (**B**) Mean intensity analysis for fluorescence signal was also performed for the images, and plotted using Graph pad prism. ** *p* < 0.01.

Polymeric nanoparticles have shown great promise as cellular drug delivery vehicles [57]. Nanoparticles could efficiently enhance drug stability, availability, and retention at the target intracellular site of action. Nevertheless, one of the important criteria that dictates the therapeutic efficacy of drug-loaded nanoparticles is the cellular uptake by target cells. In this study, therefore, we assessed the cellular uptake of Lfd-NPs by the THP-1 cell line using Rhodamine-6-G (R6G) dye. As shown in Figure 5, Lfd-NPs were efficiently taken up by THP-1 cells, as manifested by higher fluorescence signals (Figure 5A), compared to control cells. In addition, fluorescence intensity analysis (Figure 5B) revealed a higher uptake in the cells treated with dye-loaded NPs in comparison to those treated with free dye, as well as the unstained controls. This enhancement could have positive implications in terms of enhanced drug delivery, resulting in the improved therapeutic efficacy of Lfd-NPs.

#### 3.5.2. In Vitro Biocompatibility

The safety and/or biocompatibility of drug-loaded polymeric nanoparticles is an important criterion that governs the clinical applicability of NPs in the biomedical field. Nanoparticles should exert minimal toxicity and/or detrimental effects on normal off-target cells. The biocompatibility of the Lfd-NPs was evaluated on L929 murine fibroblast cells, wherein the cells were treated with different concentrations (5 to 1000 ng/mL) of Lfd-NPs. As shown in Suppl. Figure S1, no remarkable cytotoxic effect of Lfd-NPs was observed in L929 murine fibroblast cells upon treatment with Lfd-NPs, with the cell viability of the L929 murine fibroblast cells rising to more than 85%, even upon treatment with the highest dose of Lfd-NPs (100 ng/mL). These results suggest the safety and biocompatibility of the Lfd-NPs against normal off-target cells. 

#### 3.5.3. Effect of Lfd-NPs on Cytokine Production 

**Figure 6:**
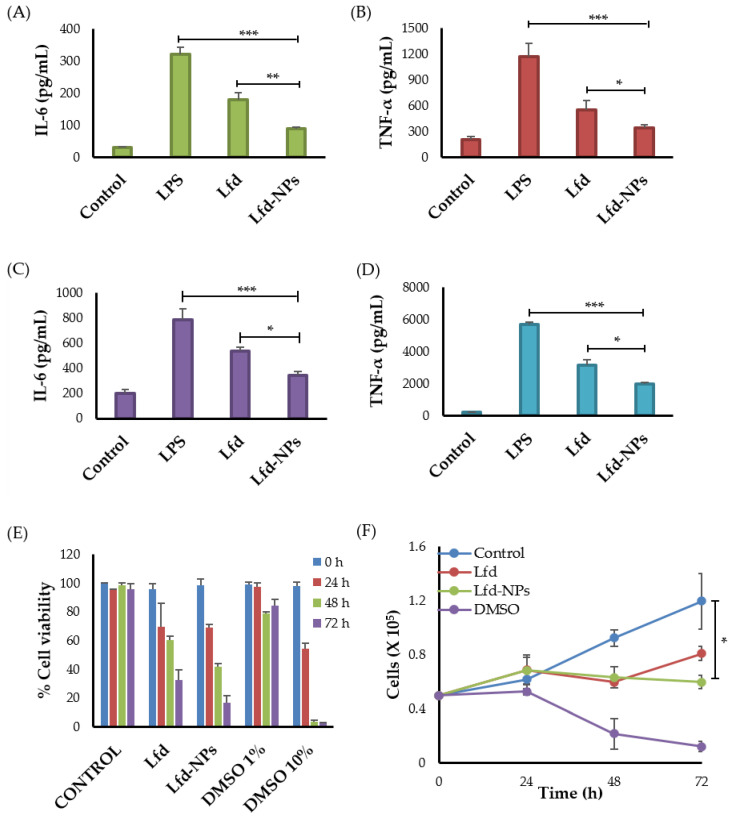
Cytokine production by THP-1 human monocytes and RAW 264.7 murine macrophage cells. LPS-treated THP-1 human monocytes and RAW 264.7 murine macrophages were treated with free Lfd or Lfd-NPs for 24 h, then (**A**) IL-6 and (**B**) TNF-α production by THP-1 human monocytes, or (**C**) IL-6 and (**D**) TNF-α production by RAW 264.7 murine macrophage cells, was assessed by ELISA. (**E**) Cell viability and (**F**) anti-proliferative activity of Jurkat T cells were assessed in response to treatment with free Lfd or Lfd-NPs. Untreated cells and cells treated with DMSO 1% and DMSO 10% were taken as controls. * *p* < 0.05, ** *p* < 0.01 and *** *p* < 0.001.

The effect of Lfd-NPs on the expression of tumor necrosis factor alpha (TNF-α) and Interleukin (IL)-6 in the supernatant of LPS-treated THP-1 human monocytes and RAW 264.7 murine macrophage cells was investigated via an enzyme-linked immunosorbent assay (ELISA) (Figure 6). TNF-α has been linked to a variety of signaling processes inside cells that result in necrosis or apoptosis [58], while IL-6 is known to contribute to an acute phase immune response via inducing the synthesis of acute phase proteins such as CRP [59]. As shown in Figure 6, in cultures of LPS-treated cells, both TNF-α and IL-6 concentrations were significantly increased, compared to untreated control cells (Figure 6A–D). LPS is widely known to enhance pro-inflammatory cytokines, such as IL-6 and TNF-α; anti-inflammatory cytokines; and chemokines cytokine production in many cell lines [58,60]. Of interest, treatment with either free Lfd or Lfd-NPs significantly reversed the induction of the pro-inflammatory cytokines (TNF-α and IL-6), compared to LPS-treated cells (Figure 6A–D). The release of TNF-α and IL-6 was significantly decreased in both cells upon treatment with Lfd-NPs compared to free Lfd. These results might be attributed to the enhanced cellular uptake of Lfd-NPs by both THP-1 human monocytes and RAW 264.7 murine macrophage cells.

Besides cytokines that are mainly produced by monocytes/macrophages, such as TNF-α and IL-6, T lymphocytes were reported to participate in the production of some inflammatory cytokines in rheumatoid arthritis [61]. Furthermore, it has been reported that leflunomide could also exhibit anti-proliferative and immune-suppressive activities against T lymphocytes [62]. Accordingly, the anti-proliferative efficacy of Lfd-NPs was investigated against Jurkat T cells. As shown in Figure 6E, both free Lfd and Lfd-NPs significantly reduced the proliferation of Jurkat T cells in a time-dependent manner; treatment of Jurkat T cells with either free Lfd or Lfd-NPs for 72 h significantly reduced cell viability to 35% and 13%, respectively, compared to an untreated control (*p* < 0.5) (Figure 6F). These results suggest that Lfd-NPs could induce their anti-inflammatory effect via suppressing cytokine release from not only monocytes/macrophages, but T lymphocytes as well.

### 3.6. In Vivo Studies

#### 3.6.1. Adjuvant-Induced Arthritis (AIA) Rodent Model

Among the various established animal models of RA, the adjuvant-induced arthritis (AIA) model is considered one of the most commonly used standard models, reflecting a number of clinical characteristics of RA in humans, such as joint inflammation and swelling, synovial tissue proliferation, and cartilage and bone destruction [61]. In this study, standard-specific visible and biochemical RA markers were taken into consideration for monitoring inflammation and disease progression, and were used for the assessment of treatment efficacy, including paw and knee swelling, locomotion behavior, limb redness and body weight loss, levels of the serum cytokines TNF-α, IL-6, and IL-1β, acute phase protein (CRP) and rheumatoid factor (RF) induction levels, and joint histopathology. Visible markers were monitored over the course of the entire study; however, biochemical and histological assessments were performed post-euthanization of the animals. 

##### Effects of Lfd-NPs on Paw and Knee Swelling

**Figure 7:**
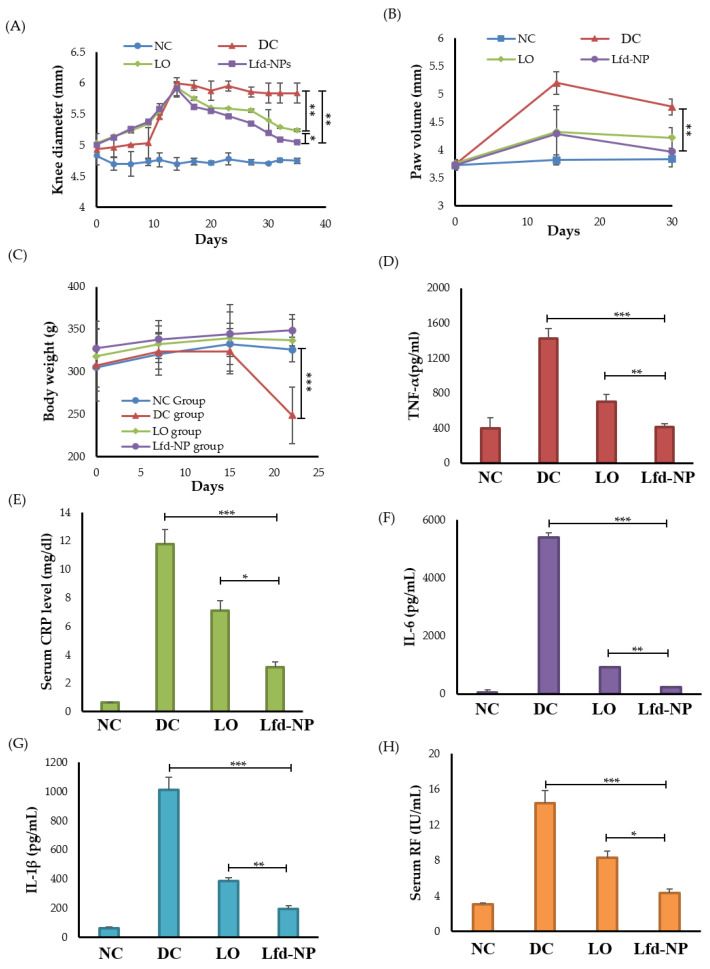
In vivo efficacy of Lfd-NPs in AIA rodent model. AIA rats were treated with either 10 mg/kg of an oral suspension of commercial leflunomide (LO; daily) or intra-articular injection of Lfd-NPs (Lfd-NP; once weekly), then treatment efficacy was evaluated. (**A**) knee diameter, (**B**) paw edema, and (**C**) body weight post-induction at different time intervals. (**D**–**F**) Analysis of serum levels of inflammatory cytokines TNF-α, IL-1β, and IL-6, respectively. (**G**) Analysis of serum C-reactive protein and (**H**) serum RF in AIA-treated rats. * *p* < 0.05, ** *p* < 0.01 and *** *p* < 0.001.

The adjuvant-induced arthritis rodent model was induced via the intra-articular injection of Complete Freund’s Adjuvant (CFA) in the right knee of rats. On day 0 of treatment, all CFA-injected animals showed obvious knee swelling, with slight redness in some rats, indicating that the AIA model was established successfully. Treatment with either oral Lfd (LO) or the intra-articular injection of Lfd-loaded nanoparticles (Lfd-NPs) significantly reduced knee swelling, compared to the DC group (Figure 7A). In addition, the intra-articular injection of Lfd-NPs resulted in a significant reduction in knee diameter, compared to animals treated with LO. Similarly, Vernier caliper readings for the measurement of paw swelling demonstrated a remarkable increase in the paw volume of all CFA-injected groups as compared to untreated (NC) rats. A significant reduction in mean paw swelling was observed in both Lfd-NP and LO groups, compared to the CFA-injected untreated (DC) group, in a treatment-duration dependent manner (Figure 7B). Among, the two groups, an effective reduction in paw swelling was observed in Lfd-NP rats more than LO rats. These results demonstrated the efficacy of Lfd-NPs in relieving RA knee and paw swelling.

##### Effects of Lfd-NPs on the Body Weight

Another important consideration is the lower food intake, and consequent body weight loss, witnessed in experimental models or clinical situations characterized by discomfort and/or limited locomotion. In the untreated DC group, remarkable body weight loss was observed and became significantly different than other groups, especially after starting Lfd treatment (Figure 7C). No significant differences in the animals’ body weight were observed among healthy untreated (NC) groups and the animals receiving either LO or Lfd-NPs. These results suggest that treatment with Lfd-NPs is well tolerated with a good safety profile.

##### Effects of Lfd-NPs on Inflammation Biomarkers and Biochemical Indicators

Inflammatory cytokines have crucial roles in the key processes associated with rheumatoid arthritis, such as joint inflammation and articular degeneration. In RA, key pro-inflammatory cytokines such as IL-1β, IL-6, and TNF-α are primarily involved. IL-1β, which can be detected in the joint cavity, has an essential role in the development of RA pathogenesis via stimulating bone resorption [63]. Similarly, TNF-α serve a key role in the pathophysiological processes of RA. TNF-α overexpression in RA can result in local joint tissue damage as well as clinical symptoms [64]. High levels of TNF-α were reported to be observed in the serum as well as synovial fluid of RA patients [65]. IL-6 is another key pro-inflammatory cytokine that is expressed abundantly in the synovial fluid and serum of patients with RA. The dysregulated persistent production of IL-6 has been associated with disease activity and joint destruction [66]. Accordingly, in this study, the impact of Lfd-NPs on serum levels of TNF-α, IL-1β, and IL-6 was evaluated. As shown in Figure 7D–F, the detected levels of all tested inflammatory cytokines were significantly higher in AIA untreated (DC) rats compared to healthy untreated (NC) rats. Both LO and Lfd-NPs efficiently reduced IL-1β, IL-6, and TNF-α levels compared with the DC group. Of interest, Lfd-NPs efficiently reduced serum levels of TNFα, IL-1β, and IL-6 to a negative control level, suggesting the possible efficacy of Lfd-NPs in treating RA-associated inflammation.

Another approach that can be used to clinically monitor the efficacy of anti-rheumatic therapy is to detect the serum levels of biochemical parameters associated with RA, namely the C-reactive protein (CRP) and rheumatoid factor (RF). Herein, serum levels of CRP and RF were measured following either LO or Lfd-NP treatments. As depicted in Figure 7G,H, the serum levels of both CRP and RF were significantly increased in AIA-untreated rats, compared to the negative control (NC) group. Treatment with either LO or Lfd-NPs significantly reduced the levels of the biochemical parameters evaluated. Of interest, serum levels of CRP and RF were significantly lower in the LNP group compared to the LO group (*p* < 0.05).

##### Histopathological Examination of Knee Joints

**Figure 8:**
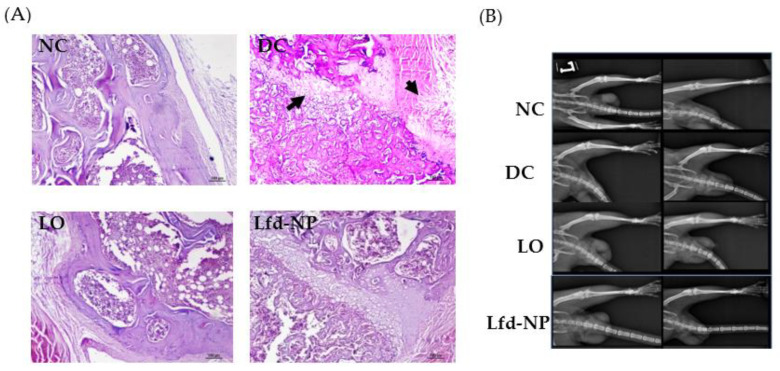
(**A**) Histopathological analysis of isolated joint tissues. (**B**) X-ray analysis of left limb. Left and right column of the X-ray panels indicate images taken before and after treatment for each group from top to bottom [NC: Normal Control, DC: Diseased control, Lfd-NP: Treated with Lfd-NPs, LO: Treated with Oral Lfd].

In addition to biochemical evidences, joint histological staining was conducted to ascertain the efficacy of different treatments. As shown in Figure 8A, no gross anomaly was observed in the morphology in the joint tissues of the NC, LO, and Lfd-NP groups, compared to the DC group. In addition, a remarkable reduction in joint tissue inflammation was observed in the Lfd-NP-treated group, compared to the LO group.

X-ray analysis did not reveal significant differences in the arthritic damage observed in terms of bone or cartilage erosion (Figure 8B). However, differences in limb swelling were observed among different groups; more swelling was inferred in the untreated DC group, compared to other treated groups. However, no significant differences in limb swelling were observed among treated groups. 

##### In Vivo Toxicity

**Figure 9:**
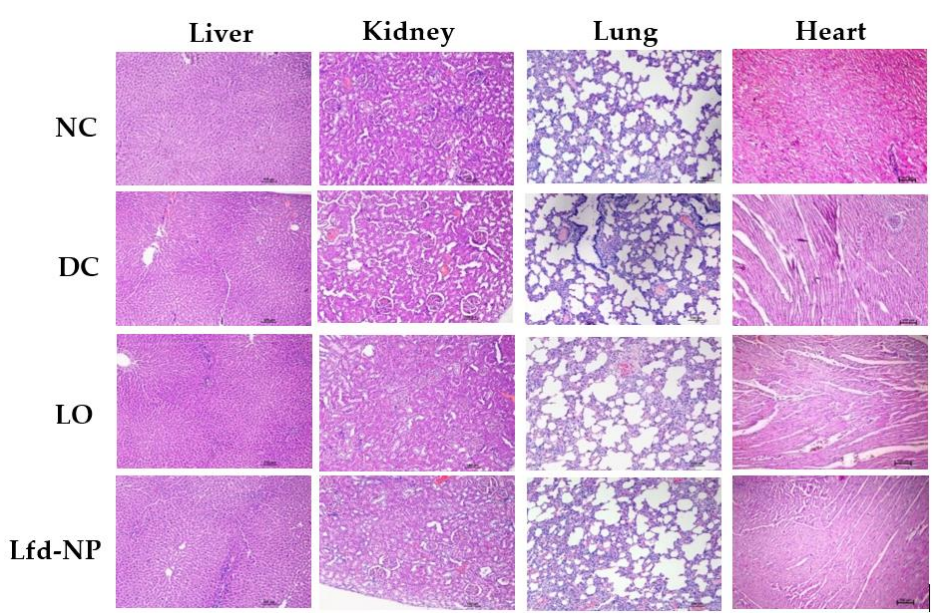
In vivo toxicity of Lfd-NPs. AIA rats were treated with either oral suspension of Lfd or intra-articular injection of Lfd-NPs. At the end of treatment, animals were euthanized and vital organs (liver, kidney, lung, and heart) were collected and investigated for signs of toxicity.

To evaluate the in vivo safety of Lfd-NPs, vital organs, including the liver, kidney, lung, and heart, were collected from animals at the end of the experiment, and histological analysis was performed. No gross morphological anomaly or toxicity was observed in any of these organs among the control and treated groups (Figure 9). Leflunomide is reported to be associated with nephrotoxicity and hepatotoxicity [67]; however, in this study, the intra-articular administration of Lfd-loaded NPs did not induce any structural differences in the homogenous organization of hepatocytes, or any degenerative alterations. No signs of necrosis or changes in the connective tissue or extracellular matrix were observed in the Lfd-NP group. Additionally, no haemorrhage and/or inflammatory infiltrate were detected. Furthermore, no changes indicative of toxicity or renal injury were identified in the kidneys of the animals.

Collectively, based on in vivo results, it was inferred that Lfd-loaded nanoparticles (Lfd-NPs) administered by an intra-articular route were effective in reducing local, as well as systemic, inflammatory signs compared to oral Lfd, with the consequent repairment of rheumatoid arthritis. Such superior efficacy of Lfd-NPs, compared to orally administered Lfd, against the AIA animal model might be attributed to the effective retention of the drug within the joint space, accompanied with long-lasting drug release in the articular joint. Consequently, the intra-articular administration of Lfd-NPs might improve intra-articular retention time, along with minimizing drug-associated systemic side effects.

## 4. Conclusions

In this study, leflunomide-loaded poly(ε-caprolactone) nanoparticles (Lfd-NPs) were successfully developed by a bottom-up approach. The developed NPs displayed an encapsulation efficiency of 79.83 ± 4.1%, and sustained drug release for up to 168 h. In addition, the in vitro anti-inflammatory activity of Lfd-NPs against differentiated and LPS-induced THP1 human monocytes revealed a remarkable down-regulation in various cytokine levels, such as TNF-α and IL-6. Most importantly, in vivo studies on the adjuvant-induced arthritis (AIA) rodent model indicated a significantly higher efficacy of weekly intra-articularly administered Lfd-NPs, compared to the daily oral dosing of commercial leflunomide. Local therapy with Lfd-NPs offered the chance for decreasing the systemic exposure of Lfd, along with prolonging drug retention time in the joint for the efficient regression of the inflammation triggered by the adjuvant. To sum up, intra-articularly administered Lfd-NPs might represent a potential therapeutic alternative to systemically administered drugs for the treatment of rheumatoid arthritis, without eliciting systemic adverse effects. Furthermore, poly(ε-caprolactone) polymeric nanoparticles might represent a promising delivery vehicle for the long-term local intra-articular administration of various immune modulator drugs and growth factors used for the treatment of rheumatoid arthritis.

## Figures and Tables

**Table 1:** Full factorial design data showing independent variables and their level of variation.

Independent Variables	Code	Level of Variation		
		−1	0	1
PCL concentration (mg)	A	20	110	200
PVA concentration (% *w*/*v*)	B	0.5	2	3.5

**Table 2:** Composition of different formulae of Lfd-NPs.

Formulation Code	Independent Variables		Response Variables		
	A (mg)	B (%)	R1 (nm)	R2 (%)	R3 (%)
F-1	0	0	242 ± 16.4	81.29 ± 2.1	23.65 ± 0.92
F-2	−1	−1	228 ± 27.6	69.61 ± 2.1	20.25 ± 1.72
F-3	−1	0	169 ± 19.5	74.43 ± 1.8	21.65 ± 2.39
F-4	−1	1	234 ± 13.4	72.33 ± 1.5	21.04 ± 2.22
F-5	1	−1	714 ± 17.3	66.83 ± 3.2	19.44 ± 2.13
F-6	1	1	754 ± 11.9	67.92 ± 2.7	19.76 ± 2.18
F-7	0	−1	363 ± 21.2	76.11 ± 2.4	22.14 ± 1.13
F-8	0	1	367 ± 13.3	78.55 ± 1.9	22.85 ± 1.74
F-9	1	0	633 ± 22.1	70.67 ± 2.3	20.56 ± 3.29

## Data Availability

Not applicable.

## Supplementary Materials

The following supporting information can be downloaded at: https://www.mdpi.com/article/10.3390/pharmaceutics14030519/s1. Figure S1: Cytotoxic effect of Lfd-NPs against L929 murine fibroblast cells upon treatment with different concentration (5 ng–1 mg) of Lfd-NPs for 48 h; Table S1: Composition of simulated synovial fluid (pH 7.4).
