# Supplementary material for: Poly ε-Caprolactone Nanoparticles for Sustained Intra-Articular Immune Modulation in Adjuvant-Induced Arthritis Rodent Model
Source: Pharmaceutics. 2022 Feb 26;14(3):519. doi: 10.3390/pharmaceutics14030519 (PMC8953799; doi:10.3390/pharmaceutics14030519)
Supplement: Supplementary file 1 [file pharmaceutics-14-00519-s001.zip › pharmaceutics-1604533-supplementary.pdf]

Supplementary Table S1. Simulated Synovial Fluid (pH 7.4).

Constituent	Concentration (g/L)
Sodium chloride (NaCl)	8.0
Potassium chloride (KCl)	0.2
Sodium phosphate dibasic (Na_2HPO_4)	1.44
Potassium phosphate monobasic (KH_2PO_4)	0.24
Hyaluronic acid	3.0

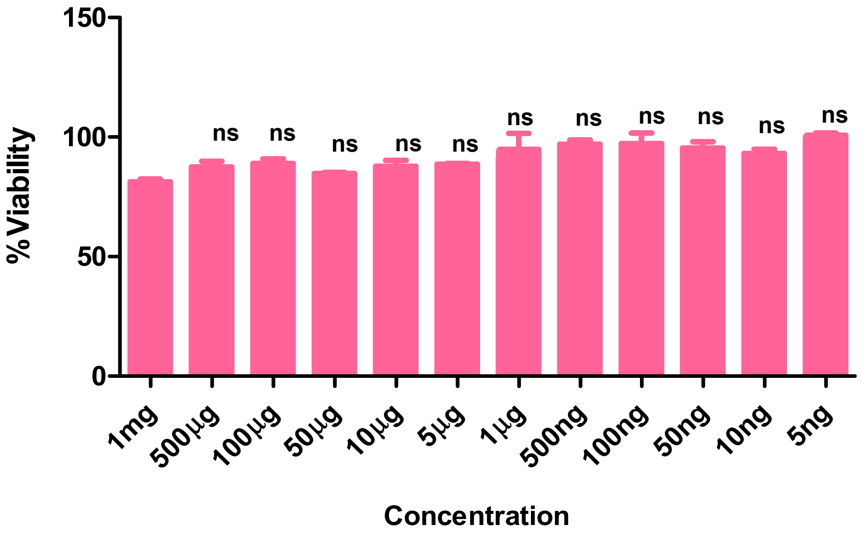

Supplementary Figure S1. Cytotoxic effect of Lfd-NPs against L929 murine fibroblast cells upon treatment with different concentration (5 ng–1 mg) of Lfd-NPs for 48 h.
